# MODE: Minimax Optimal Deterministic Experiments for Causal Inference in the Presence of Covariates

**DOI:** 10.3390/e26121023

**Published:** 2024-11-26

**Authors:** Shaohua Xu, Songnan Liu, Yongdao Zhou

**Affiliations:** 1 National Key Laboratory of Intelligent Tracking and Forecasting for Infectious Diseases (NITFID), School of Statistics and Data Science, Nankai University, Tianjin 300071, China; shxu@mail.nankai.edu.cn; 2 School of Mathematical Sciences, Inner Mongolia University, Hohhot 010021, China; liusongnan1994@hotmail.com; 3 College of Science, China University of Petroleum (East China), Qingdao 266580, China

**Keywords:** average treatment effect, covariate balance, generalized discrepancy, quasi-Monte Carlo

## Abstract

Data-driven decision-making has become crucial across various domains. Randomization and re-randomization are standard techniques employed in controlled experiments to estimate causal effects in the presence of numerous pre-treatment covariates. This paper quantifies the worst-case mean squared error of the difference-in-means estimator as a generalized discrepancy of covariates between treatment and control groups. We demonstrate that existing randomized or re-randomized experiments utilizing Monte Carlo methods are sub-optimal in minimizing this generalized discrepancy. To address this limitation, we introduce a novel optimal deterministic experiment based on quasi-Monte Carlo techniques, which effectively minimizes the generalized discrepancy in a model-independent manner. We provide a theoretical proof indicating that the difference-in-means estimator derived from the proposed experiment converges more rapidly than those obtained from completely randomized or re-randomized experiments using Mahalanobis distance. Simulation results illustrate that the proposed experiment significantly reduces covariate imbalances and estimation uncertainties when compared to existing randomized and deterministic approaches. In summary, the proposed experiment serves as a reliable and effective framework for controlled experimentation in causal inference.

## 1. Introduction

In recent years, data-driven decision-making has been routinely employed in various contexts, including evaluating the efficacy of medical treatments, vaccinations, training programs, marketing campaigns, and other interventions. The crux of effective decision-making lies in the ability to manage uncertainty and make informed choices based on available information. The concept of entropy, which quantifies uncertainty in a probability distribution, plays a crucial role in understanding how information is generated and utilized in these contexts.

Suppose an experimenter has collected data on *n* units, along with baseline covariate information. The question arises: how should treatments be allocated based on these pre-treatment covariates to provide precise causal estimates of treatment effects on potential outcomes? Here, the balance of information—essentially the reduction of uncertainty—across treatment groups becomes vital for drawing valid conclusions.

Randomized experiments, including completely randomized experiments and re-randomized experiments [1], are commonly employed as control methods. However, ref. [2] argued that a deterministic experiment can strictly dominate all randomized experiments in minimizing estimation error when continuous covariates are present. The ability of a deterministic design to minimize covariate imbalance reflects its potential to reduce uncertainty in treatment effect estimates, thereby leading to more reliable causal inferences. This deterministic experiment seeks to balance the covariate distribution across treatments, resulting in fairer experiments relative to random assignments. Consequently, deterministic experiments often appear more appealing in practice.

Ref. [2] developed a Bayesian optimal deterministic experiment (BODE) by minimizing the expected mean squared error (MSE) risk of the estimated treatment effect across assumed stochastic processes for the potential outcomes. However, identifying a BODE requires specifying approximate prior distributions for the potential outcomes, which introduces additional uncertainty in the modeling process.

In this paper, we introduce a novel type of optimal deterministic experiment, termed the minimax optimal deterministic experiment (MODE). The design criterion to be minimized is the maximum MSE risk associated with treatment assignment over a class of mean and variance functions of potential outcomes. This model-independent approach inherently reduces uncertainty by avoiding reliance on specific parametric assumptions. The bias component of the maximum risk is proportional to the generalized discrepancy, which quantifies covariate imbalance by measuring the difference between the empirical distributions of covariates in treatment and control groups. This generalized discrepancy serves as a critical measure of information loss, providing insight into the uncertainty surrounding treatment assignments.

Quasi-Monte Carlo methods are employed to generate the MODE. From a covariate balance perspective, the covariates in a randomized experiment can be viewed as a Monte Carlo sample from the covariate population. As a result, existing randomized experiments are sub-optimal, and the proposed MODE exhibits reduced covariate imbalance and lower estimation MSE. Typically, the MODE decreases the MSE of the difference-in-means estimator based on the completely randomized experiments (CRE) from $O_{p}\left(n^{-1}\right)$ to $o(n^{-1})$ when potential outcomes are fixed. Thus, for most online control experiments prioritizing estimation accuracy and the effective management of uncertainty, the proposed MODE is preferred. Additionally, we provide re-randomization relaxation of the MODE for situations where randomization is necessary due to practical constraints.

The remainder of this paper is organized as follows. In Section 1.1, we review related experimental designs for causal inference. Our main contributions are summarized in Section 1.2. In Section 2, we formulate the problem of finding a MODE. Section 3 derives the large sample properties of the MODE. Section 4 theoretically compares the MODE with existing randomized experiments. Section 5 compares the finite sample performance of the MODE with that of existing experiments across different potential outcomes models. Finally, we conclude this paper and discuss future works in Section 6. For clarity, all proofs are provided in Appendix B.

### 1.1. Related Works

Completely randomized experiments are considered the gold standard for estimating causal effects, as randomization balances all potential confounding variables—both observed and unobserved—on average, thereby providing reliable estimates of treatment effects. However, as highlighted by [3], in a particular randomized experiment, covariates between treatment groups may become significantly unbalanced, leading to increased estimation error and greater uncertainty about the treatment effects. As pointed out by [1], with 10 independent covariates, the probability of at least one covariate showing a significant difference at the 5% significance level between treatment and control groups is about 40%. Similarly, ref. [4] emphasized that achieving covariate balance (e.g., through matching) enhances robustness to varying parametric assumptions.

Numerous methods exist in the design stage of an experiment to address covariate imbalance, including blocked randomization [5] and re-randomization [1,6]. Blocking is a classical approach that is effective for balancing a limited number of discrete covariates. In contrast, re-randomization, as proposed by [1], offers a comprehensive framework for reducing covariate imbalance, thereby enhancing the reliability of causal estimates. In this method, the randomized experiment is repeated until the covariates between treatment groups meet a pre-specified balance criterion. Thus, re-randomization generalizes blocking to accommodate multiple covariates with various values. For an in-depth review of the design and analysis of randomized experiments, please refer to [7].

However, from the perspective of statistical decision-making, randomized experiments are dominated by deterministic experiments [2]. Recent works by [8,9] have introduced optimal deterministic experiments based on assumed parametric potential outcome models. The BODE proposed by [2] depends on the chosen prior for potential outcomes, which adds another layer of uncertainty. All of these optimal designs are sensitive to the specified potential outcome models. In contrast, the proposed MODE is model-independent, thereby mitigating the uncertainty associated with model specification.

The generalized discrepancy utilized in the MODE is analogous to the optimization objective of kernel optimal matching [10], which is employed for covariate balancing in the analysis stage of an experiment. However, the generalized discrepancy is applied during the design stage, while the optimization variables in kernel optimal matching are the weights for the estimator. Unlike post hoc methods, the MODE is implemented entirely in the design phase, ensuring it remains unaffected by outcome data. More convincing reasons for why as much as possible should be done in the design phase of an experiment can be found in [11].

### 1.2. Main Contributions

Firstly, we introduce a novel type of model-independent deterministic experiment, termed the MODE, which minimizes uncertainty in treatment effect estimation. Secondly, we provide a theoretical proof demonstrating that the proposed MODE reduces covariate imbalance and the estimation MSE of the completely randomized experiment or the re-randomization experiment using Mahalanobis distance, decreasing it from $O_{p}\left(n^{-1}\right)$ to $o(n^{-1})$ when potential outcomes are fixed. The third contribution of this paper is establishing the relationship between the maximum risk of causal estimators and the generalized discrepancy, emphasizing how reducing this discrepancy enhances the overall information quality in causal inference.

## 2. Problem Setups

Let $D={\left\{\left(T_{i},X_{i},Y_{i}\right)\right\}}_{i=1}^{n}$ denote the observable data for a population of *n* units, where $T_{i}$ is a binary treatment for the *i*-th unit, i.e., the *i*-th unit is treated if and only if $T_{i}=1$. $X_{i}$ and $Y_{i}$ represent the *p*-dimensional pre-treatment covariates and the real-valued outcome for the *i*-th units, respectively. Let $X={\left\{X_{i}\right\}}_{i=1}^{n}$ represent all the *p*-dimensional covariates. We define $n_{1}=\sum_{i=1}^{n}T_{i}$ and $n_{0}=\sum_{i=1}^{n}\left(1-T_{i}\right)$ as the numbers of treated and untreated units, respectively. The set $T_{n,n_{1}}=\left\{\left(T_{1},\cdots ,T_{n}\right)\in {\left\{0,1\right\}}^{n},\sum_{j=1}^{n}T_{j}=n_{1}\right\}$ includes all possible treatment assignments with $n_{1}$ treated units.

Under the finite population potential outcomes framework and the stable unit-treatment-value assumption [12], we have $Y_{i}=T_{i}Y_{i}\left(1\right)+\left(1-T_{i}\right)Y_{i}\left(0\right)$, where $Y_{i}\left(1\right)$ and $Y_{i}\left(0\right)$ are the potential outcomes for the *i*-th unit under the treatment and control, respectively. This paper considers the randomness arising from both treatment assignment and potential outcomes. For t∈{0,1} and i,j∈[n]≜{1,2⋯,n}, we assume that the conditional mean and covariance of potential outcomes given all covariates are as follows:

(1) $$\begin{array}{cc} E(Y_{i}\left(t\right)|X) & =f_{t}\left(X_{i}\right);\\ \mathrm{cov}(Y_{i}\left(t\right),Y_{j}\left(t\right)|X) & =\sigma_{t}^{2}\left(X_{i}\right)I\left\{i=j\right\};\\ \mathrm{cov}(Y_{i}\left(0\right),Y_{j}\left(1\right)|X) & =0, \end{array}$$

where $f_{t}\left(\cdot \right)$ and $\sigma_{t}^{2}\left(\cdot \right)$ are unknown mean and variance functions, respectively, and I{·} is the indicator function. The conditions in (1) impose restrictions on the correlations between potential outcomes and covariates, and they hold if $(X_{i},Y_{i}\left(0\right),Y_{i}\left(1\right)),i\in \left[n\right]$ are mutually independent [10].

Typically, the target of interest is to effectively estimate the average treatment effect

(2) $$\tau =n^{-1}\sum_{i=1}^{n}E\left(Y_{i}\left(1\right)-Y_{i}\left(0\right)|X\right),$$

using the difference-in-means estimator

(3) $$\hat{\tau }=n_{1}^{-1}\sum_{j=1}^{n}T_{j}Y_{j}-n_{0}^{-1}\sum_{j=1}^{n}\left(1-T_{j}\right)Y_{j}.$$

In this paper, we aim to design a deterministic treatment assignment $T\in T_{n,n_{1}}$ with $1\leq n_{1}\leq n-1$ to improve the estimation precision of the difference-in-means estimator. For simplicity, let $\delta =(f_{1}\left(x\right),f_{0}\left(x\right),\sigma_{1}^{2}\left(x\right),\sigma_{0}^{2}\left(x\right))$ represent the vector of mean and variance functions. The conditional MSE of $\hat{\tau }$ given T can be decomposed as follows:

$$\mathrm{MSE}\left(\hat{\tau }|T,X,\delta \right)=E\left\{{\left(\hat{\tau }-\tau \right)}^{2}|T,X,\delta \right\}=V\left(T,X,\delta \right)+B\left(T,X,\delta \right)$$

where the conditional variance and squared bias are given by the following:

(4) $$\begin{array}{cc} V(T,X,\delta ) & =n_{1}^{-2}\sum_{j=1}^{n}T_{j}\sigma_{1}^{2}\left(X_{j}\right)+n_{0}^{-2}\sum_{j=1}^{n}\left(1-T_{j}\right)\sigma_{0}^{2}\left(X_{j}\right), \end{array}$$

(5) $$\begin{array}{cc} B(T,X,\delta ) & ={\left(n_{1}^{-1}\sum_{j=1}^{n}T_{j}f_{1}\left(X_{j}\right)-n_{0}^{-1}\sum_{j=1}^{n}\left(1-T_{j}\right)f_{0}\left(X_{j}\right)-\tau \right)}^{2}. \end{array}$$

From the above expression, the MSE risk depends on the treatment assignment T, the set of covariates X, and the vector of unknown functions δ. Therefore, to minimize the MSE, we can carefully design T by fully utilizing the information contained in X and δ. Different assumptions regarding δ lead to distinct optimal treatment assignments [8,9]. However, these assumptions are unverifiable since the potential outcomes are unobservable. In this paper, we adopt a minimax framework to identify the most robust treatment assignment with respect to a class of unknown mean and variance functions. We do not impose specific functional forms on these unknown functions; instead, we restrict them to belong to a function class Δ, as rigorously defined in (7). The maximum risk of a treatment is quantified by the worst-case MSE for that treatment over all possible true mean and variance functions in Δ, i.e.,

(6) $$R\left(T\right)=\max_{\delta \in \Delta }\mathrm{MSE}\left(\hat{\tau }|T,X,\delta \right).$$

The most robust treatment assignment with respective to Δ is the one for which the maximum risk achieves the minimum over all possible treatments in $T_{n}≜\cup_{n_{1}=1}^{n-1}T_{n,n_{1}}$. More precisely, we define the MODE as follows.

**Definition 1.**  A treatment $T^{*}$ is called a MODE if $R\left(T^{*}\right)=\min_{T\in T_{n}}R\left(T\right).$

Therefore, the task of obtaining a MODE can be viewed as a game between the experimenter, who chooses a treatment assignment T from $T_{n}$, and nature, which chooses the worst-case potential outcomes structures from Δ. Additionally, the MODE is model-independent, as the maximum risk defined in (6) accounts for the uncertainties of all possible potential outcomes models. If there are multiple treatments with the minimum maximum risk, the MODE in Definition 1 is not unique, but they are equivalent in terms of minimizing the maximum risk. Therefore, we can choose any one of them to conduct the experiment.

## 3. Minimax Optimal Deterministic Experiments

In this section, we first derive two equivalent expressions for the maximum risk in (6). We then consider the MODE, as outlined in Definition 1, under both fixed and divergent sample sizes. Additionally, we provide an algorithm for generating the MODE.

### 3.1. Expressions for the Maximum Risk

For any $T\in T_{n,n_{1}}$ with $1\leq n_{1}\leq n-1$, let $X_{1}=\left\{X_{j}|T_{j}=1,j\in \left[n\right]\right\}$ and $X_{0}=\left\{X_{j}|T_{j}=0,j\in \left[n\right]\right\}$ denote the covariate information of the treated and untreated units, respectively. To ensure that the maximum risk R(T) in (6) is well defined, we assume that

(7) $$\Delta =\{\left(f_{1},f_{0},\sigma_{1}^{2},\sigma_{0}^{2}\right)|f_{t}\in \Omega ,{\left(f_{t},f_{t}\right)}_{\Omega }\leq \gamma^{2},\sigma_{t}^{2}\left(x\right)\leq \sigma^{2},t\in \left\{0,1\right\},$$

where $\gamma^{2}$ and $\sigma^{2}$ are pre-specified parameters, and Ω is a reproducing kernel Hilbert space, equipped with the inner product ${\left(\cdot ,\cdot \right)}_{\Omega }:\Omega \times \Omega \to R$ and the reproducing kernel K(·,·):X×X→R. A crucial feature of this kernel is its reproducibility, meaning that ${\left(K\left(x,\cdot \right),f\left(\cdot \right)\right)}_{\Omega }=f\left(x\right)$ for any x∈X and f∈Ω; see [13,14].

After embedding the mean functions into the reproducing kernel Hilbert space Ω, we provide an analytical expression for the maximum risk by bounding the bias term B(T,X,δ) using a generalized Koksma–Hlawka inequality.

**Theorem 1.**  *For any $T\in T_{n,n_{1}}$ with $1\leq n_{1}\leq n-1$, the maximum risk $R\left(T\right)=\max_{\delta \in \Delta }\mathrm{MSE}\left(\hat{\tau }|T,X,\delta \right)$ is expressed as follows:*

$$R\left(T\right)=\sigma^{2}\left(n_{1}^{-1}+n_{0}^{-1}\right)+\gamma^{2}{\left(D_{K}\left(X_{1},X\right)+D_{K}\left(X_{0},X\right)\right)}^{2},$$

*where $\sigma^{2}$ and $\gamma^{2}$ are constants specified in *Δ*, and $D_{K}{\left(X_{t},X\right)}^{2}$ is the squared generalized discrepancy between $X_{t}$ and X, defined by*

(8) $$D_{K}{\left(X_{t},X\right)}^{2}=n^{-2}\sum_{x,y\in X}K\left(x,y\right)-2{\left(nn_{t}\right)}^{-1}\sum_{x\in X,y\in X_{t}}K\left(x,y\right)+n_{t}^{-2}\sum_{x,y\in X_{t}}K\left(x,y\right).$$

Theorem 1 shows that the variance component of the maximum risk is minimized by any balanced experiment in $T_{n,n/2}$, i.e., $n_{1}=n_{0}=n/2$. The bias component of the maximum risk is determined by the sum of the generalized discrepancies of the treated and untreated covariates. The generalized discrepancy defined in (8) can alternatively be expressed as the squared distance between the empirical distribution functions of the point sets $X_{t}$ and X, denoted as $F_{n_{t}}\left(x\right)$ and $F_{n}\left(x\right)$, respectively. Specifically, we have

$$D_{K}{\left(X_{t},X\right)}^{2}=d_{\Omega }{\left(F_{n_{t}}\left(x\right),F_{n}\left(x\right)\right)}^{2},$$

where $d_{\Omega }\left(\cdot ,\cdot \right)$ is the distance metric induced by the inner product ${\left(\cdot ,\cdot \right)}_{\Omega }$. A point set P⊂X with a small discrepancy $D_{K}\left(P,X\right)$ indicates that the empirical distributions of P and X are close in terms of the distance metric $d_{\Omega }\left(\cdot ,\cdot \right)$.

Different choices of the reproducing kernel Hilbert spaces correspond to different generalized discrepancies, resulting in varying expressions for maximum risks. We provide two widely used reproducing kernel Hilbert spaces, as described in [15,16].

**Example 1.**  *Let $\Omega =X_{2}\left({\left[0,1\right]}^{p}\right)$, which is the set of all real-valued functions on ${\left[0,1\right]}^{p}$ that have square-integrable mixed first derivatives [15]. In this context, $D_{K}\left(X_{t},X\right)$ can represent various types of discrepancies, such as the generalized $L_{2}$-discrepancy and the centered $L_{2}$-discrepancy, etc. For instance, the kernel function for the centered $L_{2}$-discrepancy is $K\left(x,y\right)=\prod_{j=1}^{p}\left(1+0.5\right|x_{j}-0.5|+0.5|y_{j}-0.5|-0.5|x_{j}-y_{j}\left|\right)$.*

**Example 2.**  *Let $\Omega =N(R^{p})$. For the explicit form of this space, refer to [16]. It has been established that the space $N(R^{p})$ contains the Sobolev space $W_{(p+1)/2,2}$ when p is even; otherwise, $N\left(R^{p}\right)=W_{(p+1)/2,2}$, where $W_{s,2}$ is the set of functions whose s-th order derivatives are square-integrable. In this context, $D_{K}\left(X_{t},X\right)$ is the energy distance between $X_{t}$ and X, with the kernel function defined as $K\left(x,y\right)=-{\left\{\sum_{j=1}^{p}{\left(x_{j}-y_{i}\right)}^{2}\right\}}^{1/2}$.*

According to the property of the generalized discrepancy defined in (8), we provide another expression for the maximum risk.

**Corollary 1.**  *The maximum risk expression in Theorem 1 is equivalent to the following:*

$$R\left(T\right)=\sigma^{2}\left(n_{1}^{-1}+n_{0}^{-1}\right)+\gamma^{2}D_{K}{\left(X_{1},X_{0}\right)}^{2}.$$

Corollary 1 indicates that the bias component of the maximum risk can be represented as the squared generalized discrepancy between the treated covariates $X_{1}$ and the untreated covariates $X_{0}$. Consequently, the closer the empirical distributions of $X_{1}$ and $X_{0}$ are, the smaller the maximum risk becomes. The generalized discrepancy $D_{K}\left(X_{1},X_{0}\right)$ defined in (8) can be equivalently expressed as a maximum mean discrepancy [17], given by the following:

$$D_{K}\left(X_{1},X_{0}\right)=\max_{\phi \in \Omega ,{\left(\phi ,\phi \right)}_{\Omega }\leq 1}\left|n_{1}^{-1}\sum_{x\in X_{1}}\phi \left(x\right)-n_{0}^{-1}\sum_{x\in X_{0}}\phi \left(x\right)\right|.$$

Thus, $D_{K}\left(X_{1},X_{0}\right)$ serves as a measure of covariate imbalance that incorporates a range of mean differences rather than focusing solely on a specific mean difference, as done with the Mahalanobis distance defined in (14). A zero generalized discrepancy implies that the covariate distributions in the treatment and control groups are identical. This distributional consistency cannot be achieved through mean matching alone. Additionally, using the equivalent expression for maximum risk in Corollary 1 enhances computational efficiency, as evaluating the maximum risk of a treatment requires calculating the value of the discrepancy function only once.

### 3.2. MODE and Asymptotic MODE

For any fixed sample size *n* and discrepancy function $D_{K}\left(\cdot ,\cdot \right)$ in (10), we identify the MODE in Definition 1 by solving the following:

(9) $$\left(X_{1}^{*},X_{0}^{*}\right)=\arg\min_{\left(X_{1},X_{0}\right)\in X_{n}}R\left(T\right),$$

where $X_{n}=\{\left(X_{1},X_{0}\right)|X_{1}\subset X,1\leq |X_{1}\left|\leq n-1,X_{0}=X\setminus X_{1}\right\}$. The treatment $T_{n}^{*}$ corresponding to the above $\left(X_{1}^{*},X_{0}^{*}\right)$ is a MODE. When *n* is small, we can obtain the MODE $T_{n}^{*}$ by enumerating all elements in $X_{n}$. However, this solution depends on the pre-specified parameters $\gamma^{2}$ and $\sigma^{2}$, and it becomes almost infeasible for large *n* values since the cardinality of $X_{n}$ grows exponentially to $2^{n}-2$. Consequently, we explore the concept of the asymptotic MODE as *n* approaches infinity. To make progress, we require that the considered discrepancy function belongs to the following set:

(10) $$D_{X}=\left\{D_{K}\left(\cdot ,X\right)|\min_{P\subset X,|P|=\alpha n,\alpha \in (0,1)}D_{K}\left(P,X\right)=o\left(n^{-1/2}\right)\right\}.$$

Notably, a Monte Carlo point set of size αn with α∈(0,1) will typically have a discrepancy of order $O_{p}\left(n^{-1/2}\right)$. In contrast, a quasi-Monte Carlo point set, also referred to as representative points and defined as the minima of a discrepancy, possesses a discrepancy of order $o(n^{-1/2})$. This low-discrepancy property supports the theoretical assertion that quasi-Monte Carlo points outperform Monte Carlo points and is fulfilled by commonly used discrepancies. For example, ref. [16] demonstrated that the *n*-point support points converge at a rate of $O(n^{-1/2}{\left(\log n\right)}^{-(1-v)/(2p)})$ for any v∈(0,1), measured by the energy distance presented in Example 2. Additionally, Theorem 1 of [18] shows that if the empirical distribution function of the covariates $F_{n}\left(x\right)$ is joint independent, the *n*-point data-driven subsamples converge at a rate of $O(n^{-1+v})$ for any v∈(0,1/2), measured by the generalized $L_{2}$-discrepancy presented in Example 1. Thus, all discrepancies in Examples (1) and (2) belong to $D_{X}$.

For any given discrepancy function in $D_{X}$, we identify an asymptotic MODE as follows.

**Theorem 2.**  For any discrepancy $D_{K}\left(\cdot ,X\right)$ in *(10)*, the balanced treatment assignment $T_{n}^{*}$ with $X_{1}^{*}=\arg\min_{X_{1}\subset X,|X_{1}|=n/2}D_{K}\left(X_{1},X\setminus X_{1}\right)$ and $X_{0}^{*}=X\setminus X_{1}^{*}$, is an asymptotic MODE, with its maximum risk given by $R\left(T_{n}^{*}\right)=\min_{T\in T_{n}}R\left(T\right)=4\sigma^{2}n^{-1}+o\left(n^{-1}\right)$, as n→∞.

Theorem 2 ensures that the variance component of the maximum risk of the asymptotic MODE is minimized, while its bias component becomes negligible compared to its variance component by minimizing the covariate imbalance, defined by the discrepancy between the treated and untreated covariates. Consequently, experimental units with non-homogeneous covariates perform comparably to those with homogeneous covariates when utilizing the asymptotic MODE.

### 3.3. Algorithm for Generating Asymptotic MODE

Based on Theorem 2, we can implement the asymptotic MODE in the following steps. Firstly, we search for n/2 points from X that have the lowest discrepancy relative to the entire covariate dataset X. This subset is denoted as $X_{1}^{*}$, and the remaining n/2 covariates are denoted as $X_{0}^{*}$, i.e., $X\setminus X_{1}^{*}$. Then, we can assign the treatment to the units with $X_{1}^{*}$ (or $X_{0}^{*}$) and the control to the units with $X_{0}^{*}$ (or $X_{1}^{*}$). The above processes are summarized in Algorithm 1.
**Algorithm 1:** MODE: minimax optimal deterministic experiment **Input:** $X={\left\{X_{i}\right\}}_{i=1}^{n}$: the covariates of *n* experimental units; K(·,·): a valid kernel function defined on X×X; *M*: the maximum allowable sampling times. **Output:** The MODE with treatment units $X_{1}^{*}$ and control units $X_{0}^{*}$.**1****if** $K\left(x,y\right)=-{\left\{\sum_{j=1}^{p}{\left(x_{j}-y_{j}\right)}^{2}\right\}}^{1/2}$ **then****2**  $X_{1}^{*}=Twin\left(X,n/2\right),$
where Twin(X,n/2) is the n/2 points obtained by the twinning method [19].**3** **else if** $K\left(x,y\right)=K(T_{X}\left(x\right),T_{X}\left(y\right))$ **then****4**   $X_{1}^{*}=\mathrm{DDS}\left(X,n/2\right)$, where DDS(X,n/2) is the n/2 points obtained by the data-driven subsampling method [18].**5** **else****6**   **for** *m=1:M* **do****7**    $X_{1m}=SRS\left(X,n/2\right),D_{m}=D_{K}\left(X_{1m},X\setminus X_{1m}\right)$.**8**   **end****9**   $m^{*}=\arg\min_{m=1:M}D_{m},X_{1}^{*}=X_{1m^{*}}$.**10** **end****11** $X_{0}^{*}=X\setminus X_{1}^{*}$.

This algorithm systematically selects treatment and control groups to minimize covariate imbalance, thereby enhancing the robustness of treatment assignment. Any valid discrepancy on X×X can be used as the input for Algorithm 1. The optimality of the MODE is guaranteed by Theorems 2 and 3, provided the selected discrepancy belongs to $D_{K}$.

We tailor different optimization methods to various discrepancy functions. For continuous covariates, we recommend using the energy distance [16] or the empirical *F*-discrepancy [18]. When employing the energy distance as the discrepancy, characterized by the kernel $K\left(x,y\right)=-{\left\{\sum_{j=1}^{p}{\left(x_{j}-y_{j}\right)}^{2}\right\}}^{1/2}$, we recommend utilizing the twinning method proposed by [19] to identify $X_{1}^{*}$. For the empirical *F*-discrepancy using the kernel $K\left(x,y\right)=K(T_{X}\left(x\right),T_{X}\left(y\right))$, where $T_{X}\left(x\right)={\left(F_{1}\left(x_{1}\right),\cdots ,F_{p}\left(x_{p}\right)\right)}^{T}$, $F_{j}\left(x_{j}\right)$ is the empirical distribution function of the *j*-th component of X, and K(·,·) is any valid kernel on ${\left[0,1\right]}^{p}\times {\left[0,1\right]}^{p}$, we recommend the data-driven subsampling method proposed by [18] to find $X_{1}^{*}$. For the details of the twinning and the data-driven subsampling methods in Algorithm 1, please refer to [19] and [18], respectively. For discrete covariates, the Lee-discrepancy [20] is preferred. For other valid discrepancies, a Monte Carlo approximation method is applied. This involves randomly sampling *M* subsets from X and selecting the one, $X_{1}^{*}$, with the minimum discrepancy value. Ref. [2] demonstrated that after M=500 times sampling, the probability that the output $X_{1}^{*}$ is better then 99% of all possible candidates is already larger than 99%, and significantly larger values of *M* are also feasible in practice.

**Example 3.**  *This example demonstrates the usefulness of the MODE procedure using the red wine quality dataset from the UCI databases library. We evaluate the impact of treatments, such as a new storage method, on red wine quality. Two key physicochemical properties, "pH” and “alcohol,” which may influence the final outcome, are treated as covariates. We assume that all these covariates of the red wines have been collected. To minimize the influence of covariates on the estimation of treatment effects, it is essential to ensure that the covariates of the treatment and control groups are as similar as possible.*

**Figure 1:**
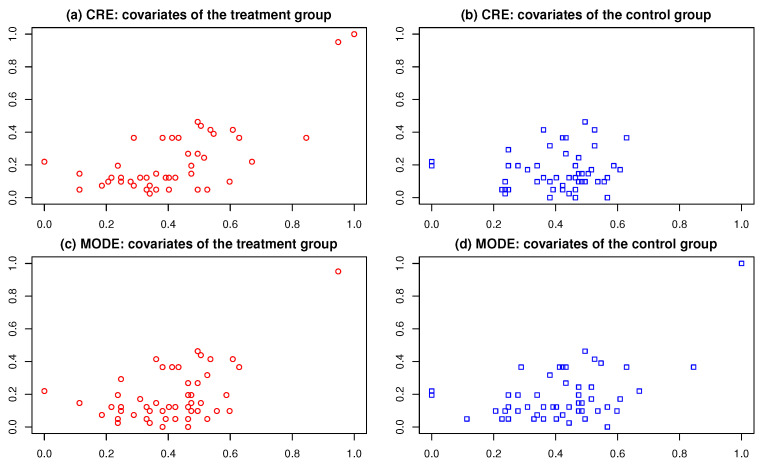
Covariates of the treatment group and the control group under the CRE (**a**,**b**) and the MODE (**c**,**d**).

*For clarity, we focus on the first 100 samples in the dataset, treating them as experimental units, and scale their covariates into the range ${\left[0,1\right]}^{2}$. Given that the covariates are continuous, we adopt the energy distance with the kernel function $K\left(x,y\right)=-{\left\{\sum_{j=1}^{p}{\left(x_{j}-y_{j}\right)}^{2}\right\}}^{1/2}$ in Algorithm 1. Figure 1 illustrates the covariates of equally sized treatment and control groups divided by the CRE and MODE methods. Intuitively, the covariates of the treatment and control groups created by MODE appear more balanced than those by CRE, particularly near the (1,1) point. Quantitatively, the Mahalanobis distance between the covariates of the treatment and control groups under MODE is 0.05, significantly smaller than the 3.41 observed with CRE. This demonstrates that MODE provides a more effective approach for achieving covariate balance, which is crucial for reducing bias in treatment effect estimation.*

## 4. Theoretical Comparison with Randomized Experiments

In this section, we theoretically compare the proposed MODE with completely randomized experiments and re-randomized experiments. When the potential outcomes are fixed, the MODE reduces the MSE of the CRE or the re-randomization using Mahalanobis distance, from $O_{p}\left(n^{-1}\right)$ to $o(n^{-1})$. Thus, the proposed MODE serves as a super effective covariate balance technique.

### 4.1. Comparison with Completely Randomized Experiments

For simplicity, we denote the difference-in-means estimators based on the CRE and the MODE as ${\hat{\tau }}_{cre}$ and ${\hat{\tau }}_{mode}$, respectively. Under the CRE, where T is randomly sampled form $T_{n,n_{1}}$ with $1\leq n_{1}\leq n-1$, ${\hat{\tau }}_{cre}$ serves as an unbiased estimator for τ, i.e., $E({\hat{\tau }}_{cre}|X,\delta )=\tau$, with a conditional variance given by the following:

(11) var(τ^cre∣X,δ)=nn1−1∑T∈Tn,n1MSE(τ^cre∣T,X,δ).

The expression in (11) indicates that this randomization variance under the CRE equals the average of the conditional MSEs across treatments in $T_{n,n_{1}}$. In contrast, the MODE in (1) minimizes the maximum MSE over a class of mean and variance functions.

We define $S^{2}\left(f_{t}\right)={\left(n-1\right)}^{-1}\sum_{i=1}^{n}{\left(f_{t}\left(X_{i}\right)-{\bar{f}}_{t}\right)}^{2}$ for t∈{0,1}, and $S^{2}\left(f_{1},f_{0}\right)={\left(n-1\right)}^{-1}\sum_{i=1}^{n}{\left(\tau_{i}-\tau \right)}^{2}$, where ${\bar{f}}_{t}=n^{-1}\sum_{i=1}^{n}f_{t}\left(X_{i}\right)$ for t∈{0,1}, and $\tau_{i}=f_{1}\left(X_{i}\right)-f_{0}\left(X_{i}\right)$ for i∈[n]. The above randomization variance can be expressed more precisely as follows (see Appendix A for details):

(12) $$\begin{array}{cc} \; & \mathrm{var}\left({\hat{\tau }}_{cre}|X,\delta \right)=E_{V}\left(X,\delta \right)+V_{E}\left(X,\delta \right)\\ \; & E_{V}\left(X,\delta \right)=n_{1}^{-1}S^{2}\left(f_{1}\right)+n_{0}^{-1}S^{2}\left(f_{0}\right)-n^{-1}S^{2}\left(f_{1},f_{0}\right),\\ \; & V_{E}\left(X,\delta \right)={\left(nn_{1}\right)}^{-1}\sum_{i=1}^{n}\sigma_{1}^{2}\left(X_{i}\right)+{\left(nn_{0}\right)}^{-1}\sum_{i=1}^{n}\sigma_{0}^{2}\left(X_{i}\right). \end{array}$$

The term $E_{V}\left(X,\delta \right)$ aligns with the variance expression when the potential outcomes are fixed [21], while the term $V_{E}\left(X,\delta \right)$ quantifies the randomness stemming from the potential outcomes.

Following the percent reduction in variance proposed by [1], we define the percent reduction in MSE as the percentage by which the MODE reduces the randomization MSE of the difference-in-means estimator:

(13) $$\mathrm{PR}\left({\hat{\tau }}_{cre},{\hat{\tau }}_{mode}|X,\delta \right)=\frac{100(\mathrm{var}\left({\hat{\tau }}_{cre}|X,\delta \right)-\mathrm{MSE}\left({\hat{\tau }}_{mode}|T_{n}^{*},X,\delta \right))}{\mathrm{var}({\hat{\tau }}_{cre}|X,\delta )}$$

where $T_{n}^{*}$ is the asymptotic MODE provided in Theorem 2. It is important to note that this percent reduction in MSE depends on the covariates X and the unknown mean and variance functions in δ. Under certain mild conditions, we provide a lower bound for this percent reduction in MSE.

**Assumption** **1.** * (i) $0<\underset{n\to \infty }{lim inf}n_{1}/n\leq \underset{n\to \infty }{lim sup}n_{1}/n<1$; (ii) $n^{-1}\sum_{i=1}^{n}f_{t}{\left(X_{i}\right)}^{2}=O_{p}\left(1\right)$, $n^{-1}\sum_{i=1}^{n}\sigma_{t}^{2}\left(X_{i}\right)=O_{p}\left(1\right)$ for t∈{0,1}.*

The first condition is necessary for the existence of the difference-in-means estimator. The second condition ensures that certain moments of the covariates are bounded in probability, which is easily satisfied if the mean and variance functions are bounded.

**Theorem 3.**  *Under the mild conditions specified in Assumption 1, the percent reduction in MSE defined in *(13)* satisfies the following:*

$$\lim_{n\to \infty }\mathrm{PR}\left({\hat{\tau }}_{cre},{\hat{\tau }}_{mode}|X,\delta \right)\geq \frac{E_{V}\left(X,\delta \right)}{E_{V}\left(X,\delta \right)+V_{E}\left(X,\delta \right)}>0.$$

Theorem 3 establishes that the proposed MODE effectively reduces the MSE of the difference-in-means estimator compared to the CRE. This reduction becomes more pronounced when the term $V_{E}\left(X,\delta \right)$ defined in (12) is small, or when the term $E_{V}\left(X,\delta \right)$ defined in (12) is large. An intuitive explanation of Theorem 3 is that while the CRE balances covariates on average, the actual distributions of covariates in the treatment and control groups may remain unbalanced in a specific experiment, resulting in larger estimation variance. In contrast, the MODE carefully arranges the treated and untreated units to ensure that their covariate distributions align as closely as possible, thereby achieving a smaller MSE.

It is evident that under Assumption 1, $\mathrm{var}\left({\hat{\tau }}_{cre}|X,\delta \right)=O_{p}\left(n^{-1}\right)$. If the potential outcomes are fixed, i.e., $\sigma_{1}^{2}\left(x\right)\equiv 0$ and $\sigma^{2}\left(x\right)\equiv 0$, Theorem 2 implies that $\mathrm{MSE}\left({\hat{\tau }}_{mode}|T_{n}^{*},X,\delta \right)=o\left(n^{-1}\right)$. Thus, the MODE demonstrates superiority over the CRE.

**Corollary 2.**  *Under the mild conditions outlined in Assumption 1, if $\sigma_{1}^{2}\left(x\right)\equiv 0$ and $\sigma^{2}\left(x\right)\equiv 0$, then the MODE reduces the MSE of the CRE from $O_{p}\left(n^{-1}\right)$ to $o(n^{-1})$, i.e., $\lim_{n\to \infty }\mathrm{PR}\left({\hat{\tau }}_{cre},{\hat{\tau }}_{mode}|X,\delta_{0}\right)=100\%$ with $\delta_{0}=\left(f_{1}\left(x\right),f_{2}\left(x\right),0,0\right)$.*

Corollary 2 indicates that the convergence rate of the MSE is improved by employing the MODE compared to the CRE when the potential outcomes are fixed. This further establishes the superiority of the proposed MODE.

### 4.2. Comparison with Re-Randomized Experiments

In this subsection, we demonstrate that the proposed MODE outperforms the re-randomized experiment using Mahalanobis distance [1], referred to as ReM, in terms of minimizing the MSE. Furthermore, the proposed MODE can also be identified as a specific case of a re-randomized experiment.

The re-randomization technique enhances the performance of a randomized experiment by excluding assignments that yield unbalanced covariate distributions prior to the experiment’s initiation. Specifically, a re-randomization criterion is defined as a binary function ϕ such that a treatment assignment T belongs to the restricted randomization set if and only if ϕ(T)=1. The restricted set for ReM is defined as follows:

$$\phi_{m}\left(T\right)=I\left\{T\in T_{n,n_{1}},M\left(X_{1},X_{0}\right)\leq a\right\},$$

where *a* is a pre-specified constant, and $M(X_{1},X_{0})$ is the Mahalanobis distance between the treatment and control groups defined by the following:

(14) $$M\left(X_{1},X_{0}\right)=\frac{n_{1}n_{0}}{n}{\left({\bar{X}}_{1}-{\bar{X}}_{0}\right)}^{T}\mathrm{cov}{\left(X\right)}^{-1}\left({\bar{X}}_{1}-{\bar{X}}_{0}\right),$$

where ${\bar{X}}_{1}=n_{1}^{-1}\sum_{i=1}^{n}T_{i}X_{i}$, ${\bar{X}}_{0}=n_{1}^{-1}\sum_{i=1}^{n}\left(1-T_{i}\right)X_{i}$, and cov(X) is the sample covariance matrix. The ReM is considered balanced when $n_{1}=n/2$.

Consider the scenario where the potential outcomes are fixed and the causal effect is additive, i.e., $\sigma_{1}^{2}\left(x\right)\equiv 0,\sigma_{0}^{2}\left(x\right)\equiv 0$ and $Y_{i}\left(1\right)=Y_{i}\left(0\right)+\tau$ for i∈[n]. In this case, the balanced ReM reduces the variance of the CRE to $\left\{1-\left(1-v_{a}\right)R^{2}\right\}\mathrm{var}\left({\hat{\tau }}_{cre}|X,\delta \right)$ (Theorem 3.2 of [1]), where $v_{a}=P\left(\chi_{p+2}^{2}\leq a\right)/P\left(\chi_{p}^{2}\leq a\right)$, $\chi_{p}^{2}$ is the Chi-square distribution with *p* degrees of freedom, and $R^{2}$ represents the squared multiple correlation between ${\left\{Y_{i}\left(0\right)\right\}}_{i=1}^{n}$ and ${\left\{X_{i}\right\}}_{i=1}^{n}$. Thus, the balanced ReM enjoys the same convergence rate with the CRE. We summarize this conclusion as the following corollary.

**Corollary 3.**  *If $\sigma_{1}^{2}\left(x\right)\equiv 0,\sigma^{2}\left(x\right)\equiv 0$, $Y_{i}\left(1\right)=Y_{i}\left(0\right)+\tau$ for i∈[n], and $n^{-1}\sum_{i=1}^{n}f_{t}{\left(X_{i}\right)}^{2}=O_{p}\left(1\right)$ for t∈{0,1}, then the variance of the balanced ReM is of order $O_{p}\left(n^{-1}\right)$.*

Theorem 2 implies that $\mathrm{MSE}\left({\hat{\tau }}_{mode}|T_{n}^{*},X,\delta \right)=o\left(n^{-1}\right)$ if $\sigma_{1}^{2}\left(x\right)\equiv 0$ and $\sigma^{2}\left(x\right)\equiv 0$. Thus, as a result of Corollary 3, the proposed MODE improves the convergence rate of the estimated treatment effect compared to the balanced ReM from $O_{p}\left(n^{-1}\right)$ to $o(n^{-1})$. This further reinforces the superiority of using the proposed MODE.

Next, we establish a connection between the MODE and re-randomization. In our setting, the restricted set for the MODE, $T_{n}^{*}$, can be defined as follows:

$$\phi_{mode}\left(T\right)=I\left\{T\in T_{n,n/2},D_{K}\left(X_{1},X_{0}\right)=\min_{\left(X_{1},X_{0}\right)\in X_{n}}D_{K}\left(X_{1},X_{0}\right)\right\},$$

where $X_{n}=\{\left(X_{1},X_{0}\right)|X_{1}\subset X,1\leq |X_{1}\left|\leq n-1,X_{0}=X\setminus X_{1}\right\}$. The unknown minimum value of the discrepancy in this re-randomization criterion can be estimated via Monte Carlo sampling. Specifically, we can randomly sample from $T_{n,n/2}$ a feasible number of times and take the minimum discrepancy among those samples as its estimate.

To leverage the strengths of randomized-based inference, we observe that the restricted set $\phi_{mode}\left(T\right)$ typically comprises either singletons or sets of small cardinality. Therefore, we relax the re-randomization criterion $\phi_{mode}\left(T\right)$ to the following:

(15) $$\phi_{mode,\alpha }\left(T\right)=I\left\{T\in T_{n,n/2},D_{K}\left(X_{1},X_{0}\right)\leq {\hat{q}}_{D_{K},\alpha }\right\},$$

where ${\hat{q}}_{D_{K},\alpha }$ is the empirical α-quantile of the distribution of all discrepancy values over $X_{n}$. It is evident that the above re-randomization, $\phi_{mode,\alpha }\left(T\right)$, degenerates into the MODE as α→0 and into the CRE as α→1. Thus, the re-randomized relaxation in (15) with α∈(0,1) effectively combines the randomness of the CRE with the efficiency of the MODE, making it useful when the experimenter is constrained to use randomization due to practical considerations.

## 5. Simulations

In this section, we compare the performance of several experimental approaches, including the balanced CRE, the balanced ReM utilizing the critical value $a=\chi_{p,0.2}^{2}$ [1], the BODE with default priors as outlined by [2], and the proposed MODE employing the energy distance with the kernel function $K\left(x,y\right)=-{\left\{\sum_{j=1}^{p}{\left(x_{j}-y_{j}\right)}^{2}\right\}}^{1/2}$. Each experiment is repeated 1000 times across all settings. The simulation results indicate that the proposed MODE demonstrates reduced covariate imbalance and lower estimation uncertainty compared to existing methods.

### 5.1. Covariate Imbalance

We examine two sample size and dimensionality scenarios: (i) n=1000 and p=5; (ii) n=2000 and p=10. In each case, the covariates are generated as $X_{ij}\overset{i.i.d.}{∼}U\left[-3,3\right]$ and $X_{ij}\overset{i.i.d.}{∼}N\left(0,3\right)$ for i∈[n],j∈[p]. We then apply the four experimental designs to the covariate set $X={\left\{{\left(X_{i1},\cdots ,X_{ij}\right)}^{T}\right\}}_{i=1}^{n}$. The Mahalanobis distance $M(X_{1},X_{0})$, as defined in (14), and the energy distance $D_{K}\left(X_{1},X_{0}\right)$, which represents the discrepancy function in (8) with the kernel function $K\left(x,y\right)=-{\left\{\sum_{j=1}^{p}{\left(x_{j}-y_{j}\right)}^{2}\right\}}^{1/2}$, are employed as two measures of covariate imbalance.

**Figure 2:**
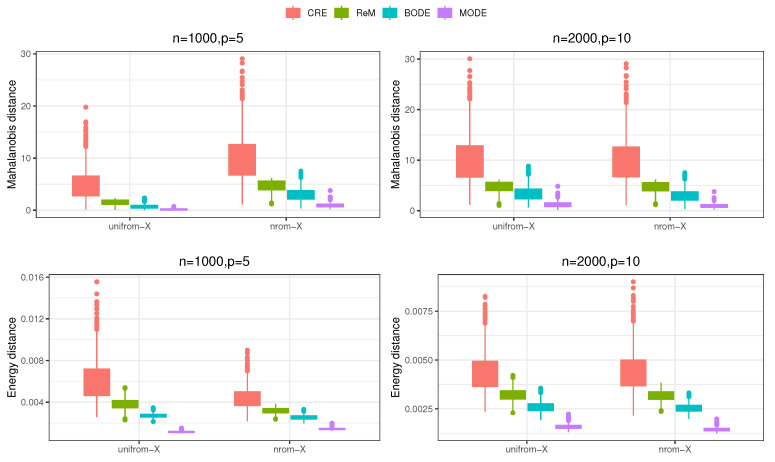
Covariate imbalances, measured based on the Mahalanobis distance and the energy distance, of various experiments under different values of *n* and *p*.

Figure 2 illustrates the covariate imbalances across the various experimental methods. The CRE method balances covariates on average; however, many CRE instances exhibit substantial covariate imbalance. In contrast, the ReM method maintains both the Mahalanobis distance and energy distance of covariates between treatment and control groups within a smaller range. The BODE further enhances covariate balance through Monte Carlo optimization. Notably, the proposed MODE demonstrates the smallest covariate imbalance across different settings. These findings suggest that the experimental units in the treatment and control groups based on MODE are more homogeneous, resulting in more comparable experimental outcomes.

### 5.2. Estimation Precision

To evaluate the precision of difference-in-means estimators derived from various experiments, we assume that the responses $Y_{1},\cdots ,Y_{n}$ are generated from the following potential outcomes model:

$$\left\{\begin{array}{cc} \; & Y_{i}=T_{i}Y_{i}\left(1\right)+\left(1-T_{i}\right)Y_{i}\left(0\right),i\in \left[n\right];\\ \; & Y_{i}\left(1\right)=f_{1}\left(X_{i}\right)+\sigma_{1}\left(X_{i}\right)u_{i},i\in \left[n\right];\\ \; & Y_{i}\left(0\right)=f_{0}\left(X_{i}\right)+\sigma_{0}\left(X_{i}\right)v_{i},i\in \left[n\right];\\ \; & \left\{X_{ij},i\in \left[n\right],j\in \left[p\right]\right\}\overset{i.i.d.}{∼}U\left[-3,3\right];\\ \; & \left\{u_{i},i\in \left[n\right]\right\}\overset{i.i.d.}{∼}N\left(0,1\right),\left\{v_{i},i\in \left[n\right]\right\}\overset{i.i.d.}{∼}N\left(0,1\right); \end{array}\right.$$

where $T_{i}=1$ if and only if the *i*-th unit is treated in the corresponding experiment. We consider four different specifications for mean and variance functions:

C1. $f_{1}\left(x\right)=e^{\tilde{x}},f_{0}\left(x\right)=1+\tilde{x},\sigma_{1}^{2}\left(x\right)\equiv 0,\sigma_{0}^{2}\left(x\right)\equiv 0$;
C2. $f_{1}\left(x\right)=e^{\tilde{x}},f_{0}\left(x\right)=1+\tilde{x},\sigma_{1}^{2}\left(x\right)\equiv 1,\sigma_{0}^{2}\left(x\right)\equiv 1$;
C3. $f_{1}\left(x\right)=e^{\tilde{x}}+e^{\tilde{x}/2},f_{0}\left(x\right)=e^{\tilde{x}}-e^{\tilde{x}/2},\sigma_{1}^{2}\left(x\right)\equiv 0,\sigma_{0}^{2}\left(x\right)\equiv 0$;
C4. $f_{1}\left(x\right)=e^{\tilde{x}}+e^{\tilde{x}/2},f_{0}\left(x\right)=e^{\tilde{x}}-e^{\tilde{x}/2},\sigma_{1}^{2}\left(x\right)\equiv 1,\sigma_{0}^{2}\left(x\right)\equiv 1$,

where $\tilde{x}=\sum_{j=1}^{p}x_{j}/\sqrt{p}$. In C1 and C3, we assume that the potential outcomes are fixed.

Table 1 presents the empirical MSEs of different-in-means estimators across the various experiments. As the sample sizes increase, the empirical MSEs decrease, as expected; however, the percentage reductions in MSE of the MODE compared to the CRE become more pronounced, indicating that the MSEs associated with the MODE converge more rapidly. Under each configuration of dimension and sample size, the ReM reduces the randomization variance of the CRE by constraining the Mahalanobis distances of the CREs. The BODE generally yields a smaller MSE than the ReM. Notably, the proposed MODE achieves the smallest MSE across various scenarios. Furthermore, the percentage reduction in MSE of the MODE over the CRE is particularly significant when the potential outcomes are fixed (C1 and C3), consistent with the optimality outlined in Corollary 2.

We also compare the differences between the true distribution of potential outcomes and the empirical distributions of the observed outcomes derived from various experiments. Table 2 presents the Kolmogorov–Smirnov values $\sup_{y\in R}\left|F\left(y\right)-F_{n}\left(y\right)\right|$ for the different experiments, where F(y) and $F_{n}\left(y\right)$ represent the empirical distribution functions of the potential and observed outcomes from the treatment group, respectively.

As anticipated, all Kolmogorov–Smirnov values decrease, indicating that the observed outcomes converge to the true potential outcomes in distribution as the sample sizes increase. Across all settings, the MODE yields the lowest Kolmogorov–Smirnov values, suggesting that the observed outcomes are closer to the true potential outcomes in distribution. This property of matching distributions, which benefits from quasi-Monte Carlo techniques, also enhances the estimation of treatment effects beyond the average treatment effect.

## 6. Conclusions and Discussion

This paper introduces a novel measure of covariate imbalance, termed the generalized discrepancy, and proposes a MODE designed to minimize this discrepancy. Both theoretical analysis and simulations demonstrate that the proposed MODE outperforms existing methods, such as CRE and ReM, in terms of minimizing MSE. Thus, the MODE serves as a super-effective framework for controlled experimentation in the presence of covariates.

Importantly, the covariate imbalance, defined as the generalized discrepancy, quantifies the differences in covariate distributions between treatment and control groups. This measure can also be utilized in designing experiments aimed at improving the accuracy of estimating various causal effects, such as quantile treatment effects and average treatment effects on the treated group. Such applications enable researchers to evaluate the impacts of interventions more comprehensively.

For most control experiments where estimation accuracy is the primary objective, such as online A/B tests, the proposed MODE is the preferred method. Since MODE is deterministic, randomization inference based on the MODE is limited [22]; however, asymptotic inference remains feasible. In situations where exact inference is the primary goal or randomization is necessary due to practical constraints—such as policy requirements, ethical considerations, or the presence of potential unobserved confounding variables—we recommend using re-randomization based on the MODE described in (15). This approach randomizes a set of treatments that are near the minima of the maximum risk, thus enhancing covariate balance.

The MODE can be directly extended to multi-level experiments by sequentially minimizing generalized discrepancies across treatment groups. Thus, it offers a potential alternative to the OSAT tool [23] for sample-to-batch allocation in genomics experiments. The MODE proposed in this paper assumes that all covariates are collected in advance of conducting the experiment, including scenarios such as online A/B tests, classrooms with student data, or companies with employee records. Another potential future research direction involves adaptive allocation, particularly in biomedical experiments with human subjects, where treatments must be assigned either individually or in batches as participants are enrolled.

## Figures and Tables

**Table 1:** Empirical MSEs ($\times 10^{4}$) for difference-in-means estimators based on 1000 replications across various experiments. The columns labeled PR indicate the percentage reduction in MSE of the MODE compared to the CRE. The rows labeled C1–C4 represent the four specifications of the potential outcomes model.

	CRE	ReM	BODE	MODE	PR	CRE	ReM	BODE	MODE	PR
	p=5,n=250					p=5,n=1000				
C1	620	254	124	57	91%	129	62	36	8	94%
C2	623	347	211	142	77%	148	77	55	30	80%
C3	1212	652	389	149	88%	272	165	101	18	93%
C4	1496	771	451	235	84%	301	171	119	38	87%
	p=10,n=500					p=10,n=2000				
C1	508	311	273	70	86%	118	68	70	11	91%
C2	539	374	312	112	79%	133	88	76	22	83%
C3	1374	1087	904	226	84%	321	236	213	34	90%
C4	1466	1117	916	265	82%	306	253	217	44	86%

**Table 2:** Average Kolmogorov–Smirnov values ($\times 10^{2}$) for potential and observed outcomes from the treatment group, based on 1000 replications of various experiments. The rows labeled C1–C4 correspond to the four specifications of the potential outcomes model.

	CRE	ReM	BODE	MODE	CRE	ReM	BODE	MODE
	p=5,n=250				p=5,n=1000			
C1	5.07	4.47	4.02	3.30	2.57	2.39	2.03	1.56
C2	5.29	5.00	4.68	4.41	2.64	2.49	2.40	2.38
C3	5.45	4.62	3.92	3.14	2.61	2.35	2.12	1.61
C4	5.21	4.67	4.46	4.30	2.70	2.47	2.34	2.21
	p=10,n=500				p=10,n=2000			
C1	3.74	3.44	3.19	2.93	1.92	1.70	1.58	1.34
C2	3.80	3.65	3.39	3.47	1.93	1.72	1.73	1.69
C3	3.68	3.38	3.21	2.88	2.00	1.67	1.55	1.37
C4	3.68	3.62	3.38	3.22	1.88	1.83	1.73	1.61

## Data Availability

The R codes for generating the figures and tables in this paper are available at the following website: https://github.com/Kids1997/R-packages/blob/main/MODE (accessed on 6 November 2024).

## Abbreviations

The following abbreviations are used in this manuscript:

BODE	Bayesian Optimal Deterministic Experiment
CRE	Completely Randomized Experiment
MODE	Minimax Optimal Deterministic Experiment
MSE	Mean Squared Error
ReM	Re-randomized Experiment using Mahalanobis distance

## Appendix A. The Randomization Variance for ${\hat{\tau }}_{cre}$

To derive the explicit expression for the randomization variance of ${\hat{\tau }}_{cre}$, we apply the law of total variance, which implies the following:

(A1) $$\mathrm{var}\left({\hat{\tau }}_{cre}|,X,\delta \right)=\mathrm{var}\left(E\left({\hat{\tau }}_{cre}|Y,X,\delta \right)\right)+E\left(\mathrm{var}\left({\hat{\tau }}_{cre}|Y,X,\delta \right)\right).$$

On the one hand, the first term can be computed as follows:

(A2) $$\mathrm{var}\left(E\left({\hat{\tau }}_{cre}|Y,X,\delta \right)\right)=n^{-2}\sum_{i=1}^{n}\left(\sigma_{1}^{2}\left(X_{i}\right)+\sigma_{0}^{2}\left(X_{i}\right)\right).$$

On the other hand, ref. [12] demonstrated that the conditional variance of ${\hat{\tau }}_{cre}$ given $Y={\left\{Y_{i}\right\}}_{i=1}^{n}$ is as follows:

$$\mathrm{var}\left({\hat{\tau }}_{cre}|Y,X,\delta \right)=n_{1}^{-1}S^{2}\left(1\right)+n_{0}^{-1}S^{2}\left(0\right)-n^{-1}S^{2}\left(v\right),$$

where $S^{2}\left(t\right)={\left(n-1\right)}^{-1}\sum_{j=1}^{n}{\left(Y_{i}\left(t\right)-{\bar{Y}}_{1}\left(t\right)\right)}^{2}$, $\bar{Y}\left(t\right)=n^{-1}\sum_{i=1}^{n}Y_{i}\left(t\right)$ for t∈{0,1}, $S^{2}\left(v\right)={\left(n-1\right)}^{-1}\sum_{i=1}^{n}{\left(v_{i}-v\right)}^{2}$, $v_{i}=Y_{i}\left(1\right)-Y_{i}\left(0\right)$, and $v=n^{-1}\sum_{i=1}^{n}v_{i}$. Taking the expectation with respect to Y and applying algebra yields the following:

(A3) $$\begin{array}{cc} E(\mathrm{var}\left({\hat{\tau }}_{cre}|Y,X,\delta \right))= & n_{1}^{-1}S^{2}\left(f_{1}\right)+n_{0}^{-1}S^{2}\left(f_{0}\right)-n^{-1}S^{2}\left(f_{1},f_{0}\right)\\ \; & +{\left(nn_{1}\right)}^{-1}\sum_{i=1}^{n}\sigma_{1}^{2}\left(X_{i}\right)+{\left(nn_{0}\right)}^{-1}\sum_{i=1}^{n}\sigma_{0}^{2}\left(X_{i}\right)\\ \; & -n^{-2}\sum_{i=1}^{n}\left(\sigma_{1}^{2}\left(X_{i}\right)+\sigma_{0}^{2}\left(X_{i}\right)\right). \end{array}$$

The desired expression for the randomization variance is obtained by substituting Equations (A2) and (A3) into Equation (A1).

## Appendix B. Proofs

**Proof of Theorem 1.** Note that the bias term in (5) can be equivalently expressed as follows:

$$B\left(T,X,\delta \right)={\left(Q\left(f_{1},X_{1},X\right)-Q\left(f_{0},X_{1},X\right)\right)}^{2},$$

where $Q\left(f_{t},X_{t},X\right)=n_{1}^{-1}\sum_{x\in X_{t}}f_{t}\left(x\right)-N^{-1}\sum_{x\in X}f_{t}\left(x\right)$ represents the average difference of $f_{t}\left(\cdot \right)$ across the sets $X_{t}$ and X for t∈{0,1}. We define the linear operator L:Ω→R as $Lf=Q\left(f,X_{t},X\right)=\int f\left(x\right)d\left[F_{n_{t}}-F_{n}\right]\left(x\right)$, where $F_{n_{t}}\left(x\right)$ and $F_{n}\left(x\right)$ are the empirical distribution functions of the sets $X_{t}$ and X. It is evident that L is a bounded operator. Based on the Riesz representation theorem, there exists a unique representer ξ∈Ω satisfying $Lf={\left(f,\xi \right)}_{\Omega }$ for all f∈Ω. Let $K_{u}\left(v\right)=K\left(u,v\right)$. Due to the symmetry and reproducing properties of the kernel K(·,·), we have $\xi \left(u\right)={\left(\xi \left(\cdot \right),K_{u}\left(\cdot \right)\right)}_{\Omega }=LK_{u}\left(\cdot \right)=\int K_{x}\left(u\right)d\left[F_{n_{t}}-F_{n}\right]\left(x\right)$. Now, consider the following:

$$\begin{array}{cc} {\left(\xi ,\xi \right)}_{\Omega } & ={\left(\int K_{x}\left(\cdot \right)d\left[F_{n_{t}}-F_{n}\right]\left(x\right),\int K_{y}\left(\cdot \right)d\left[F_{n_{t}}-F_{n}\right]\left(y\right)\right)}_{\Omega }\\ \; & =\int \int {\left(K_{x}\left(\cdot \right),K_{y}\left(\cdot \right)\right)}_{\Omega }d\left[F_{n_{t}}-F_{n}\right]\left(x\right)d\left[F_{n_{t}}-F_{n}\right]\left(y\right)\\ \; & =\int \int K\left(x,y\right)d\left[F_{n_{t}}-F_{n}\right]\left(x\right)d\left[F_{n_{t}}-F_{n}\right]\left(y\right)\\ \; & =n^{-2}\sum_{x,y\in X}K\left(x,y\right)-2{\left(nn_{t}\right)}^{-1}\sum_{x\in X,y\in X_{t}}K\left(x,y\right)+n_{t}^{-2}\sum_{x,y\in X_{t}}K\left(x,y\right)\\ \; & =D_{K}\left(X_{t},X\right). \end{array}$$

Consequently, the Cauchy–Schwarz inequality implies that

$$|Q\left(f_{t},X_{t},X\right)|=|Lf_{t}\left|=\right|{\left(f_{t},\xi \right)}_{\Omega }|\leq ∥f_{t}{∥}_{\Omega }{∥\xi ∥}_{\Omega }={∥f_{t}∥}_{\Omega }D_{K}\left(X_{t},X\right),\;\mathrm{for}\;t\in \left\{0,1\right\}.$$

Thus, for any δ∈Δ, the bias term $B\left(T,X,\delta \right)\leq \gamma^{2}{\left(D_{K}\left(X_{1},X\right)+D_{K}\left(X_{0},X\right)\right)}^{2}$ with equality if and only if $\left(f_{1}\left(x\right),f_{0}\left(x\right)\right)\equiv \gamma \left(\pm \xi \left(x\right),\mp \xi \left(x\right)\right)$. The variance term $V\left(T,X,\delta \right)\leq \sigma^{2}\left(n_{1}^{-1}+n_{0}^{-1}\right)$ with equality if and only if $\sigma_{t}^{2}\left(x\right)\equiv \sigma^{2}$ for t∈{0,1}. This establishes the expression for the maximum risk. □

**Proof of Corollary 1.** It is evident that $X_{1}=X\setminus X_{0}$ and $X_{0}=X\setminus X_{1}$. According to Proposition 1 of [19], we have $D_{K}\left(X_{1},X\right)=\left(n_{0}/n\right)D_{k}\left(X_{1},X_{0}\right)$ and $D_{K}\left(X_{0},X\right)=\left(n_{1}/n\right)D_{k}\left(X_{0},X_{1}\right)$. The symmetry of K(·,·) implies that $D_{k}\left(X_{0},X_{1}\right)=D_{k}\left(X_{1},X_{0}\right)$. Thus, $D_{K}\left(X_{1},X\right)+D_{K}\left(X_{0},X\right)=D_{K}\left(X_{1},X_{0}\right)$, which completes the proof. □

**Proof of Theorem 2.** On the one hand, the expression in Theorem 1 implies that $R\left(T\right)\geq 4\sigma^{2}n^{-1}$ for any $T\in T_{n}$. On the other hand, the definition of $X_{1}^{*}$ implies that $D_{K}\left(X_{1}^{*},X_{0}^{*}\right)=2D_{K}\left(X_{1}^{*},X\right)=2\min_{P\subset X,|P|=n/2}D_{K}\left(P,X\right)=o\left(n^{-1/2}\right)$. Thus, $R\left(T_{n}^{*}\right)=4\sigma^{2}n^{-1}+o\left(n^{-1}\right)∼4\sigma^{2}n^{-1}$ as n→∞. This proves that $T_{n}^{*}$ is an asymptotic minimax optimal deterministic experiment. □

**Proof of Theorem 3.** When $\sigma_{t}^{2}\left(x\right)\equiv \sigma^{2}$ for t∈{0,1} and the conditions in Theorem 3 hold, we know that $V_{E}\left(X,\delta \right)=\sigma^{2}\left(n_{1}^{-1}+n_{0}^{-1}\right)$ and $E_{V}\left(X,\delta \right)=O\left(n^{-1}\right)$. According to Theorem 2, we have $V\left(T_{n}^{*},X,\delta \right)=4\sigma^{2}n^{-1}$, and $B\left(T_{n}^{*},X,\delta \right)=o\left(n^{-1}\right)$. Thus,

$$\begin{array}{cc} \mathrm{PR}({\hat{\tau }}_{cre},{\hat{\tau }}_{mode}|X,\delta_{0}) & =\frac{V_{E}\left(X,\delta \right)+E_{V}\left(X,\delta_{0}\right)-\left(V\left(T_{n}^{*},X,\delta \right)+B\left(T_{n}^{*},X,\delta \right)\right)}{E_{V}\left(X,\delta_{0}\right)+V_{E}\left(X,\delta_{0}\right)}\\ \; & =\frac{\left(\sigma^{2}\left(n_{1}^{-1}+n_{0}^{-1}\right)-4\sigma^{2}n^{-1}\right)+E_{V}\left(X,\delta_{0}\right)}{E_{V}\left(X,\delta_{0}\right)+V_{E}\left(X,\delta_{0}\right)}+o\left(1\right)\\ \; & \geq \frac{E_{V}\left(X,\delta_{0}\right)}{E_{V}\left(X,\delta_{0}\right)+V_{E}\left(X,\delta_{0}\right)}+o\left(1\right), \end{array}$$

which completes the proof. □
